# DEPRESSION, SOCIAL RELATIONS, AND COGNITIVE HEALTH DURING THE COVID-19 PANDEMIC

**DOI:** 10.1093/geroni/igac059.126

**Published:** 2022-12-20

**Authors:** Jasmine Cooper, Toni Antonucci, Kristine Ajrouch

**Affiliations:** University of Michigan, Ann Arbor, Michigan, United States; University of Michigan, Ann Arbor, Michigan, United States; Eastern Michigan University, Ypsilnati, Michigan, United States

## Abstract

This study examines cognitive abilities, number of people in a social network, and presence of depressive symptoms among the “youngest old”, “middle old”, and “oldest old”, by ethnicity. A sample of 600 people; 200 Blacks, 200 Whites, and 200 non-white Arabs were recruited for the survey. Those with missing data were omitted from analysis, which left 435. Data were collected via a computer assisted telephone survey. Data were analyzed using two-way ANOVAs. We found significant effects on cognition by ethnicity f(2,430)=2.71, p<0.05 and age group f(2,430)=11.24, p<0.001. There was a significant effect of the number of people in social networks on cognition across all groups f(2,430)=10.4, p<0.01. The presence of depressive symptoms was not a mediator. There was no significant interaction between ethnicity and age group, or ethnicity and social network structure.

